# Mammalian-adaptive mutation NP-Q357K in Eurasian H1N1 Swine Influenza viruses determines the virulence phenotype in mice

**DOI:** 10.1080/22221751.2019.1635873

**Published:** 2019-07-03

**Authors:** Wenfei Zhu, Zhaomin Feng, Yongkun Chen, Lei Yang, Jia Liu, Xiyan Li, Suli Liu, Lijuan Zhou, Hejiang Wei, Rongbao Gao, Dayan Wang, Yuelong Shu

**Affiliations:** a National Institute for Viral Disease Control and Prevention, Collaboration Innovation Center for Diagnosis and Treatment of Infectious Diseases, Chinese Center for Disease Control and Prevention, Beijing, People’s Republic of China; b Key Laboratory for Medical Virology, National Health and Family Planning Commission, Beijing, People’s Republic of China; c School of Public Health (Shenzhen), Sun Yat-sen University, Guangdong, People’s Republic of China

**Keywords:** Eurasian avian-like H1N1 swine influenza viruses, NP gene, NP-K357Q, pathogenicity, pandemic potential

## Abstract

It has recently been proposed that the Eurasian avian-like H1N1 (EA H1N1) swine influenza virus (SIV) is one of the most likely zoonotic viruses to cause the next influenza pandemic. Two main genotypes EA H1N1 viruses have been recognized to be infected humans in China. Our study finds that one of the genotypes JS1-like viruses are avirulent in mice. However, the other are HuN-like viruses and are virulent in mice. The molecular mechanism underlying this difference shows that the NP gene determines the virulence of the EA H1N1 viruses in mice. In addition, a single substitution, Q357K, in the NP protein of the EA H1N1 viruses alters the virulence phenotype. This substitution is a typical human signature marker, which is prevalent in human viruses but rarely detected in avian influenza viruses. The NP-Q357K substitution is readily to be occurred when avian influenza viruses circulate in pigs, and may facilitate their infection of humans and allow viruses also carrying NP-357K to circulate in humans. Our study demonstrates that the substitution Q357K in the NP protein plays a key role in the virulence phenotype of EA H1N1 SIVs, and provides important information for evaluating the pandemic risk of field influenza strains.

## Introduction

Pigs are well-known “genetic mixing vessels” for human and avian influenza viruses [1,2]. Human and avian influenza viruses can readily infect pigs, since their respiratory tracts contain receptors for both human and avian influenza viruses. It has been suggested that the past four pandemic pathogens, including the 1918 H1N1, 1957 H2N2, 1968 H3N2, and 2009 pdmH1N1, were generated in pigs [3]. Therefore, the surveillance and research on the viruses circulating in pig populations are very important in the preparedness and responses to the future influenza pandemics.

Three subtypes of influenza A viruses are currently circulating in pigs globally, the H1N1, H1N2, and H3N2 viruses [4]. In China, distinct lineages of swine influenza viruses (SIVs), including triple reassortant H1N1 (TR H1N1), Eurasian avian-like H1N1 (EA H1N1), classical swine H1N1 (CS H1N1), and reassortant H3N2 (RA H3N2), are known to co-circulate in pigs [5,6]. Since 2005, the EA H1N1 viruses have become dominant [5,6]. After the 2009 pdmH1N1 pandemic viruses transmitted back into pigs, they have reassorted continuously with EA H1N1 viruses and generated a series of variants [6–8]. After their long-term evolution, the EA H1N1 viruses in China have been reported to preferentially bind to human-type receptors, and some of the viruses tested were transmitted in ferrets by respiratory droplets. This suggests that among the influenza viruses currently circulating in animals, the EA H1N1 SIVs pose the greatest pandemic threat [6].

Swine influenza viruses could sporadically cross the species barrier and infect humans after the exposure of humans to infected pigs [9,10]. In China, the first reported human case of EA H1N1 SIV (A/Jiangsu/1/2011, JS1) infection was detected in 2011 [11]. Thereafter, six human cases of EA SIV infection were recorded, and another four viruses were isolated: A/Hebei-Yuhua/SWL1250/2012 (HB1) [12], A/Hunan/42443/2015 (HuN)[13], A/Yunnan-Longyang/SWL1982/2015 (YN1), and A/Yunnan-Wuhua/SWL1869/2015 (YN2). A full-genome analysis of these viruses showed that they can be divided into two main genotypes, represented by the JS1 and HuN viruses (Supplementary Figure 1). Full genome of JS1-like viruses (JS1 and HB1 viruses) all originated from EA SIVs [11,12], whereas the HuN-like viruses (HuN, YN1, and YN2 viruses) are reassortants of EA SIVs (HA, NA, and M genes), 2009 pdmH1N1 (PB2, PB1, PA, and NP genes), and CS H1N1 SIVs (NS gene) [13].

Several viral genetic factors are reported to contribute to the transmissibility, virulence, and pandemic potential of the influenza A viruses, or to their ability to cross species barriers. The polymerase complex genes play a critical role in the replication and adaptation of avian influenza viruses. An H5N1 virus that derived its PA or NS gene from the 2009/H1N1 virus became transmissible to guinea pigs [14]. Various mutations in the polymerase complex are also associated with increased virulence, including PB2-E158G [15], A271T [16], PB1-R198K [17–19], PB1-F2-N66S [20], PA-I127V [21], and so on. Of these, the E627K and/or D701N mutations in the PB2 protein are critical in the mammalian adaptation of avian influenza viruses [22,23]. Avian viruses with lysine at position 627 in PB2 protein replicate efficiently in human cells [24,25] and display higher pathogenicity in mice [26–29]. 701N in PB2 is involved in the increased lethality of the H5N1, H7N1, and H7N9 viruses in mice [29–33]. Both of these PB2 mutations also contribute to the transmission of the H5N1 virus in guinea pigs [27,34–37].

In our previous study, we evaluated the replication and virulence of the JS1 and HuN viruses. The HuN viruses were more virulent than the JS1 viruses in a mouse model [13]. However, the underlying mechanism was not clarified. In this study, we investigate the molecular mechanism underlying this difference in viral virulence.

## Materials and methods

### Ethics statement

The mouse studies were conducted with the approval of the Ethics Committee of the National Institute for Viral Disease Control and Prevention, China CDC (20160226008). All experiments involving live viruses were performed in a biosecurity level 2 laboratory.

### Cells

Madin–Darby canine kidney cells (MDCK), human type II alveolar epithelial (A549) cells, human embryonic kidney cells (293T), and porcine kidney cells (PK15) were maintained in Dulbecco’s modified Eagle’s medium (DMEM; Invitrogen, Carlsbad, CA, USA) supplemented with 10% foetal bovine serum (FBS; Invitrogen), HEPES (10 mM; Invitrogen), penicillin (100 units/ml), and streptomycin (100 μg/ml; Invitrogen). Cells were incubated in a humidified atmosphere of 5% CO_2_ at 37°C.

### Viruses and virus titration

The Eurasian avian H1N1 viruses, A/Jiangsu/1/2011 (JS1, H1N1) [11], A/Hebei-Yuhua/SWL1250/2012 (HB1) [12], A/Hunan/42443/2015 (HuN, H1N1) [13], A/Yunnan-Longyang/SWL1982/2015 (YN1), and A/Yunnan-Wuhua/SWL1869/2015 (YN2), were isolated from patients during 2011–2015. The viruses were titrated in MDCK cells, and the median tissue culture infective dose (TCID_50_) was calculated with the Reed–Münch formula [38].

### Plasmid construction and mutagenesis

All eight gene segments of the JS1 and HuN viruses were amplified with reverse transcription (RT)–PCR and cloned into the vRNA–mRNA bidirectional expression vector pHW2000, which was kindly provided by Professor Yi Guan, Hong Kong University. Mutations were introduced into the NP gene to generate the corresponding mutant segments. The presence of the introduced mutation and the absence of additional unwanted mutations were verified by sequencing.

### Virus rescue

All the reassortant and mutant viruses were rescued by the co-transfection of the eight reverse-genetic plasmids containing the double-stranded DNA (dsDNA) representing each gene segment into a monolayer of co-cultured 293T and MDCK cells. All the recombinant viruses were sequenced completely to ensure the absence of unwanted mutations.

### Polymerase activity assay

293T cells were co-transfected with a firefly RNP luciferase reporter plasmid, a *Renilla* luciferase expressing plasmid pRL-TK (as an internal control), and the RNP expression plasmids using SuperFect Transfection Reagent (Qiagen, Valencia, CA, USA), according to the manufacturer’s instructions. Mock transfections were performed using the two reporter constructs only. Luciferase activity was measured at 24 h after transfection, according to the Dual-Luciferase Reporter Assay kit protocol (Promega, Madison, WI, USA). The firefly RNP polymerase activity was normalized against the Renilla activity.

### Virus growth kinetics study

Confluent A549 and PK15 cells were infected with indicated recombinant viruses at the multiplicity of infection (MOI) of 0.001. The viral inoculums were removed after 1 h of adsorption and incubated at 37°C in infection medium. Supernatants were collected at 0, 12, 24, 36, 48, 60 and 72 h post infection, and viral titres were determined by TCID_50_ in MDCK cells.

### Mouse experiments

In the mouse experiments, 8–10-week-old specific-pathogen-free female C57BL/6J mice (Vital River Laboratories, Beijing, China) were used. To compare viral pathogenicity, the mice (*n* = 5) were anaesthetized with 0.2 ml of pentobarbital sodium and intranasally inoculated with 10^4^ or 10^6^ TCID_50_ of the indicated viruses in 50 μl volumes, or mock inoculated with phosphate-buffered saline (PBS). Body weights were measured daily. Mice that lost more than 30% of their original weight were euthanized for humane reasons.

To determine the morbidity and mortality in the mice, groups of mice (*n* = 5) were inoculated intranasally with the recombinant viruses at the indicated dose (10^1^∼10^6^ TCID_50_) in 50 μl volumes, or inoculated with PBS as the controls. Their body weights were measured daily. At 14 days post inoculation (dpi), serum samples were collected from the surviving mice and treated with a receptor-destroying enzyme (Denka Seiken) for 18 h at 37°C, heat-inactivated at 56°C for 30 min, and then tested for hemagglutinin inhibiting (HI) antibody titres with 0.5% (vol/vol) chicken erythrocytes. Samples with HI titres of > 40 were considered as antibody positive.

To determine the viral replication in the infected mice, groups of mice were intranasally inoculated with 10^5^ TCID_50_ of the indicated viruses and three mice in each group were euthanized at 1, 4, and 7 dpi. Respiratory tissues, including the nasal turbinate, tracheal tissue, and lungs were collected and the viral titres were measured with a TCID_50_ assay in MDCK cells.

### Phylogenetic analyses

To determine the distributions of amino acids at position 357 in the NP protein in field strains, a total of 31,614 NP amino acid sequences from the National Center for Biotechnology Information (NCBI) public database were downloaded and analysed. The sequences were first aligned with MAFFT v7.222 [39] and simplified with the cd-hit-est model. A maximum likelihood (ML) tree was constructed with MEGA 7.0[40]. The GTR substitution model assuming a gamma distribution (four gamma categories) and invariant sites was selected, and the initial tree was constructed automatically. A graphical view of the phylogenetic tree was generated with FigTree v1.4.3.

### Statistical analysis

All determinations were performed in triplicate and repeated three times. Data are expressed as means ± SD. Statistical significance was determined using non-parametric tests and the GraphPad Prism 6.0 software package (GraphPad Software Inc., San Diego, CA, USA). A *P*-value <.05 was deemed to indicate statistical significance.

## Results

### Different pathogenicity phenotypes of HuN-like and JS1-like EA SIVs in mice

**Figure 1.**
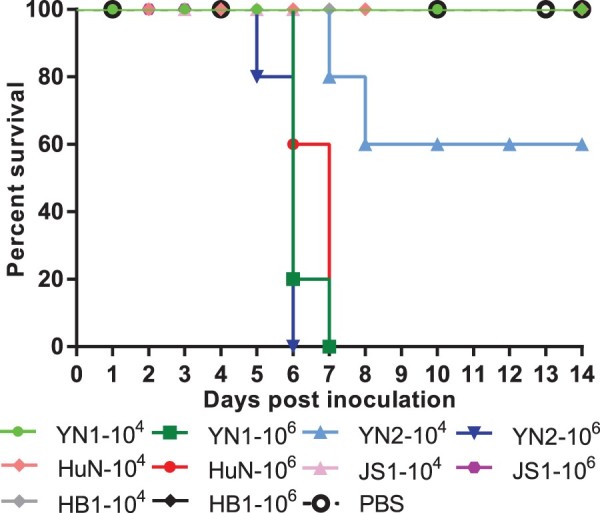
Survival rates of mice after inoculation with different viruses. Female C57BL/6J mice (8–10 weeks old) were inoculated intranasally with 50 μl of PBS containing 10^4^ TCID_50_ or 10^6^ TCID_50_ of virus A/Yunnan-Longyang/SWL1982/2015 (YN1), A/Yunnan-Wuhua/SWL1869/2015 (YN2), A/Hunan/42443/2015 (HuN), A/Jiangsu/1/2011 (JS1), or A/Hebei-Yuhua/SWL1250/2012 (HB1). Mouse bodyweights were measured daily. Mice that lost > 30% of their original bodyweight or died naturally were recorded as “dead.”

To compare the pathogenicity of the HuN-like and JS1-like EA SIVs, we infected mice (*n* = 5 per group) with each EA SIV and monitored their survival and weight loss for 14 dpi. When infected with 10^6^ TCID_50_ viruses, all the mice infected with the HuN-like viruses, including HuN, YN1, and YN2, died during the observation period. When infected with 10^4^ TCID_50_ of the HuN-like viruses, all the mice survived, except when infected with the YN2 virus, which caused 40% (2/5 mice) mortality (Figure 1). In contrast, even when infected with the highest dose (10^6^ TCID_50_) of JS1-like viruses, including the JS1 and HB1 viruses, the cohorts of mice showed 100% survival during 14 days post inoculation (Figure 1). These results suggest that these EA SIVs present different pathogenicity phenotypes in mice, and the HuN-like viruses show higher pathogenic to mice than the JS1-like viruses.

### NP gene critically determines the pathogenicity of EA SIVs

The gene cassettes of the HuN-like and JS1-like viruses differ in their internal genes (Supplementary Figure 1). To identify the genes of the HuN-like viruses responsible for their higher pathogenicity, we cloned all the gene segments of the HuN or JS1 viruses into ambisense expression plasmids and generated reassortant viruses of the HuN-WT (wild type), JS1-WT, and recombinant viruses, carrying seven segments of HuN and one segment of JS1 or seven segments of JS1 and one segment of HuN (virus nos 1–9 and 17–25, respectively, in Supplementary Table 1). Rescued viruses were inoculated into mice at indicated dosages. Both survival situation and body weight loss were recorded for 14 days.

**Figure 2.**
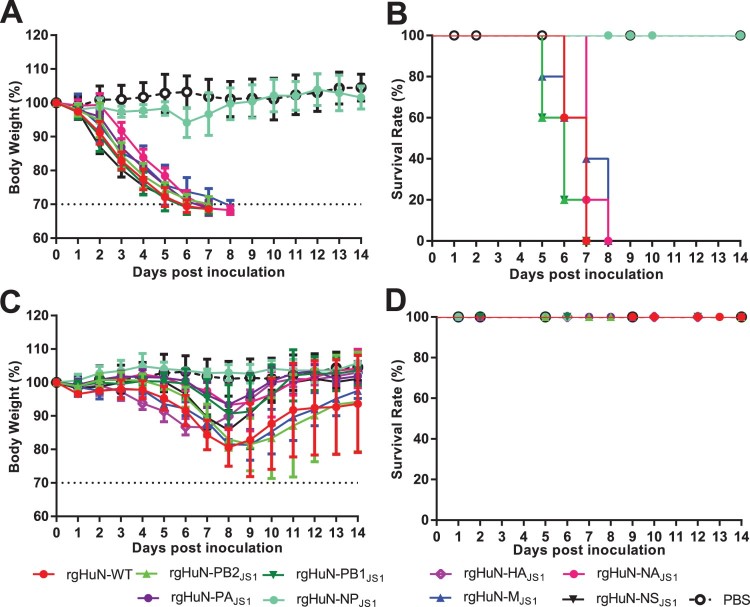
Weight changes and survival rates of mice infected with HuN reassortant viruses. The pathogenicity of reassortants was evaluated by inoculating groups of mice (*n* = 5 per group) with 10^6^ TCID_50_ (A, B) or 10^4^ TCID_50_ (C, D) of virus in 50 μl of PBS or with PBS only (control). Bodyweight loss (A, C) and death (B, D) were monitored during the 14-day observation period after inoculation. rgHuN-WT, rescued wild-type HuN virus; rgHuN-PB2_JS1_, rescued HuN virus carrying the PB2 gene from JS1 virus; rHuN-PB1_JS1_, rescued HuN virus carrying the PB1 gene from JS1 virus; rgHuN-PA_JS1_, rescued HuN virus carrying the PA gene from JS1 virus; rgHuN-NP_JS1_, rescued HuN virus carrying the NP gene from JS1 virus; rgHuN-HA_JS1_, rescued HuN virus carrying the HA gene from JS1 virus; rgHuN-NA_JS1_, rescued HuN virus carrying the NA gene from JS1 virus; rgHuN-M_JS1_, rescued HuN virus carrying the M gene from JS1 virus; rgHuN-NS_JS1_, rescued HuN virus carrying the NS gene from JS1 virus.

Compared with the rgHuN-WT virus, the body weight loss in the inoculated mice was lowest in the mice infected with rgHuN-NP_JS1,_ which carried the NP gene of JS1 and other genes of HuN (Figure 2(A,C)), although the HuN viruses carrying either segment from the JS1 virus caused more or less effect on the bodyweight loss in the mice. On the other hand, although all the mice infected with 10^4^ TCID_50_ of the recombinant viruses survived (Figure 2(D)), this was not the case when the mice were infected with 10^6^ TCID_50_ of the recombinant viruses (Figure 2(B)). All the mice inoculated with the rgHuN-NP_JS1_ virus survived, whereas all the mice infected with the other recombinant viruses died, like those infected with the rgHuN-WT virus (Figure 2(B)). Correspondingly, the NP gene of the HuN virus significantly increased the bodyweight loss and fatality rates caused by the JS1 viruses (Supplementary Figure 2(A–B)). All the mice survived when infected with wild-type (WT) JS1 viruses, whereas they all died when infected with the rgJS1-NP_HuN_ virus carrying the NP gene from the HuN virus (Supplementary Figure 2(A–B)). These results demonstrate that the NP gene is the major determinant of the pathogenicity of the HuN-like viruses.

### Substitutions R305K, F313V, and Q357K in the NP protein are mammalian signatures for the influenza A viruses

To determine the contributions of individual amino acid substitutions in more detail, we compared the NP sequences of the JS1-like and HuN-like viruses. As shown in Supplementary Table 2, a total of 35 amino acid differences were detected. A statistical analysis of the 35 sites that differed between the human, swine, and avian viruses showed that three of them are mammalian signature residues, including 305K, 313V, and 357K. HuN-like viruses have the residues 305K, 313V, and 357K, while the JS1-like viruses have the residues 305R, 313F, and 357Q. According to Supplementary Table 3, 97.8%, 97.5%, and 97.6% of the human influenza viruses carried 305K, 313V/Y, and 357K in the NP protein, respectively. In contrast, 1%, 0.1%, and 0.4% of the avian influenza viruses carried these three residues, respectively. Approximately 98.9%, 99%, 99.5% of the avian viruses encoded the JS1-like resides, 305R, 313F, and 313Q, respectively.

**Figure 3.**
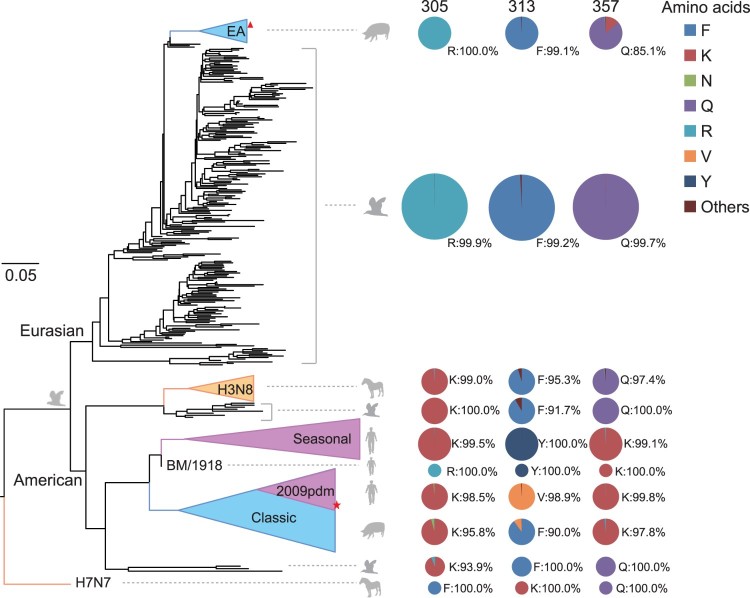
Phylogenetic analysis of NP sequences of influenza A viruses and the prevalence of different amino acids at residues 305, 313, and 357. A maximum likelihood tree of NP sequences of influenza A viruses from different host species was constructed. Clusters were generally classified into Eurasian avian-like SIVs, avian influenza viruses, horse influenza viruses H3N8, human seasonal influenza viruses, 1918 H1N1 viruses, 2009 pdmH1N1 viruses, classical SIVs, and horse influenza virus H7N7 (shown from top to the bottom in Figure 3). Pie charts indicate the proportions of different amino acids at positions 305, 313, and 357 in the NP proteins in each cluster. Red triangles indicates JS1-like viruses. Red stars indicate HuN-like viruses.

To estimate the evolutionary selection and origins of these three amino acids in animals, we analysed the NP sequences of a variety of avian and mammalian influenza viruses deposited in the NCBI and Global Initiative on Sharing All Influenza Data (GISAID) databases. As shown in Figure 3, two major branches were detected, one corresponding to the American lineage and the other to the Eurasian lineage of viruses. The major subclade in the American lineage included the classical swine and human seasonal viruses, and a minor subclade in the American lineage included equine and avian influenza viruses. The Eurasian lineage included avian and avian-like swine influenza viruses.

According to Figure 3, the distributions of amino acids at residue 305 were lineage specific. Almost all the viruses in the Eurasian lineage contained 305R (99.9%∼100%), whereas almost all those in the American lineage contained 305K (99.9%∼100%). The distributions of amino acids at residues 313 and 357 were host specific. In the avian isolates in both lineages, 313F and 357Q were dominant (Figure 3). However, in the seasonal human isolates, including subtypes H1N1, H2N2, and H3N2 (*n* = 8991) and the 1918 H1N1 viruses (*n* = 1), amino acids 313Y and 357K occurred at very high frequencies (99.1%–100%). Most of the 2009 pdmH1N1 human isolates (*n* = 5782) contained 313V and 357K (Figure 3). Intriguingly, the amino acids at residues 313 and 357 of the avian or human influenza viruses were both detected in SIVs. Most SIVs contained 313F and 357K, whereas some classical SIVs and EA-SIVs contained 313V and 357Q. These results suggest that 305K, 313V/Y, and 357K confer advantages on the viruses circulating in humans, and may facilitate the enhanced pathogenicity of the HuN-like viruses in mice.

### Substitution at NP-357 determines the pathogenicity of EA SIVs

**Figure 4.**
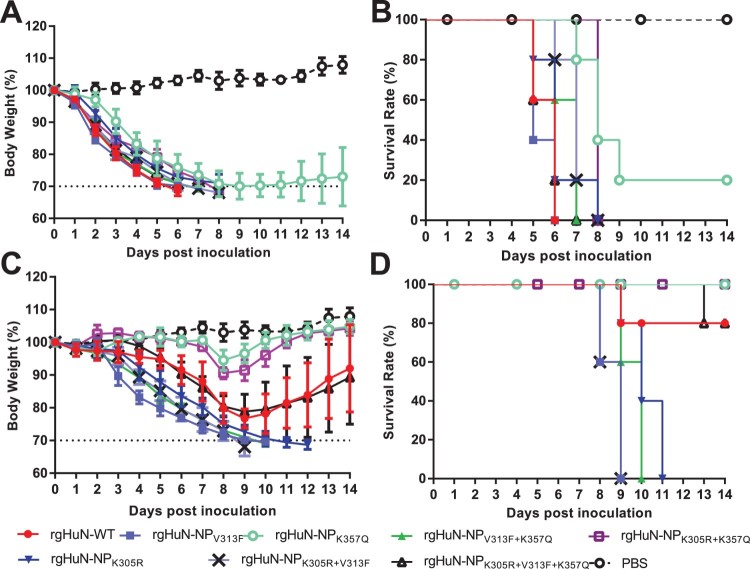
The pathogenicity of HuN mutants in mice. Mice (*n* = 5 per group) were inoculated with 10^6^ TCID_50_ (A, B) or 10^4^ TCID_50_ (C, D) of HuN mutants. Body loss (A, C) and death (B, D) were monitored for the 14 days post-inoculation. rgHuN-WT, wild-type virus carrying NP-305K, – 313V, and – 357K; rgHuN-NP_K305R_, with NP-305R, – 313V, and – 357K; rgHuN-NP_V313F_, with NP-305K, – 313F, and – 357K; rgHuN-NP_K357Q_, with NP-305K, – 313V, and – 357Q; rgHuN-NP_K305R+V313F_, with NP-305R, – 313F, and – 357K; rgHuN-NP_K305R+K357Q_, with NP-305R, – 313V and – 357Q; rgHuN-NP_V313F+K357Q_, with NP-305K, – 313F and – 357Q; rgHuN-NP_K305R+V313F+ K357Q_, with NP-305R, – 313F and – 357Q.

To further investigate the impact of NP-305K, – 313V, and/or – 357K on viral pathogenicity, recombinant viruses were generated (virus nos 10–16 and 26–32 in Supplementary Table 1). Bodyweight losses and survival rates were determined by inoculating mice with 10^4^ or 10^6^ TCID_50_ of the recombinant viruses. When inoculated with 10^6^ TCID_50_ of the HuN-backbone viruses, body weight of all the mice were dramatically reduced (Figure 4(A)). Meanwhile, all mice were dead, except for 1/5 mice infected with rgHuN-NP_K357Q_ (Figure 4(B)). When infected with 10^4^ TCID_50_ of recombinant virus, the mice infected with the wild-type HuN (rgHuN-WT) or rgHuN-NP_K305R+V313F+K357Q_ virus showed a reduction in bodyweight of up to 20% by 9 dpi, and 4/5 mice survived the infection (80% survival rate; Figure 4(C–D)). All the rgHuN-NP_K305R_-, rgHuN-NP_V313F_-, rgHuN-NP_K305R+V313F_-, or rgHuN-NP_V313F+K357Q_-infected mice became severely ill, lost bodyweight from 2 dpi (> 30% from baseline), and eventually died or were humanly euthanized by 12 dpi (0% survival; Figure 4(C–D)). In contrast, the two cohorts of mice infected with rgHuN-NP_K357Q_ or rgHuN-NP_K305R+K357Q_ showed 100% survival for 14 dpi, a very modest bodyweight loss (< 10%), and few clinical symptoms (Figure 4(C–D)). Correspondingly, the mice infected with the wild-type JS1 virus showed no obvious bodyweight loss and no mice died during the whole observation period. However, the introduction of substitution Q357K into JS1 NP resulted in significant bodyweight loss and 4/5 fatalities in the mice (Supplementary Figure 2(C–D)). These results suggest that the mutation of NP-357 enhances the pathogenicity of EA viruses in mice.

### Substitution NP-K357Q decreases the infectivity, replication, and virulence of EA SIVs

To evaluate the contribution of substitution NP-Q357K to the virulence and infectivity of EA SIVs in mammals, the 50% mouse infective dose (MID_50_) and 50% mouse lethal dose (MLD_5_) were first determined.

**Figure 5.**
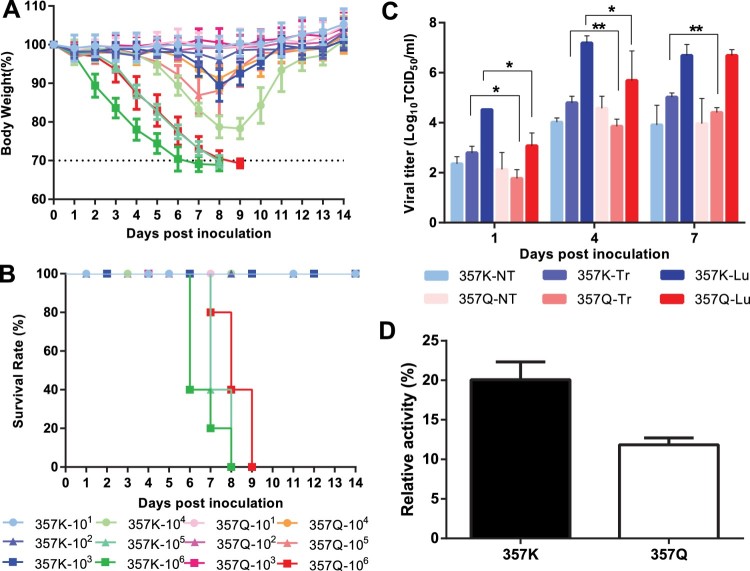
Morbidity, mortality, replication, and polymerase activity assays of rgHuN-WT (357K) and rgHuN-NP_K357Q_ (357Q) viruses in mice. Female C57BL/6J mice (8–10 weeks old) were inoculated intranasally with 50 μl of PBS containing 10^1^ TCID_50_–10^6^ TCID_50_ of the recombinant virus rgHuN-WT or rgHuN-NP_K357Q_, or with PBS (mock), to evaluate virally induced morbidity (A) and mortality (B). Morbidity was assessed by bodyweight changes over a 14-day period and was graphed as a percentage of the average weight on the day of inoculation (day 0). The average bodyweight change in each group is shown with error bars representing the standard deviations (±). Mortality associated with infection with the recombinant virus rgHuN-WT (357K) or rgHuN-NP_K357Q_ (357Q) was also examined. (C) Viral replication efficiency in the respiratory tracts of mice was analysed by inoculating mice (*n* = 3 per group per time point) intranasally with 10^5^ TCID_50_ of the recombinant viruses. At 1, 4, and 7 days postinoculation, three mice in each group were euthanized. Tissues, including nasal turbinate (NT), tracheal tissue (Tr), and lung (Lu), were collected for virus titration. (D) Polymerase assay was conducted in 293T cells transfected with reporter plasmids pFluc (encoding firefly luciferase flanked by the noncoding regions of the influenza NP segment to produce an artificial influenza-NP-like RNA segment), pRluc (pRL-TK, encoding *Renilla* luciferase, for normalization), and expression plasmids encoding PB2, PB1, PA, and NP. 357K, wild-type virus carrying NP-357K; 357Q, virus carrying NP-357Q. *p < .05; **p < .01.

When inoculated with 10^1^–10^3^ TCID_50_ of the recombinant rgHuN-WT or rgHuN_K357Q_ virus, no mice died and the bodyweight loss was < 20% (Figure 5(A)). However, when infected with 10^4^–10^6^ TCID_50_ of virus, the bodyweight loss was dramatically greater in the mice infected with rgHuN-WT (357K in the NP protein) than in those infected with rgHuN_K357Q_ (357Q in the NP protein) (Figure 5(A)). According to antibody tests, the MID_50_ value of the rgHuN-WT virus was 10^1.1^ TCID_50_, almost 20-fold lower than that of the rgHuN_K357Q_ virus (10^2.3^ TCID_50_) (Table 1). This result indicated that the substitution NP-K357Q reduced the infectivity of the EA SIVs in mice.

**Table 1:** Hemagglutinin inhibition antibody test of the C57BL/6 mice inoculated with recombinant rgHuN-WT or rgHuN-NP_K357Q_ viruses.

Inoculation viruses	Inoculation doses (log TCID_50_)	HI titres					MID_50_ (log TCID_50_)
		#1	#2	#3	#4	#5	
**rgHuN-WT**	6	ND	ND	ND	ND	ND	1.1
	5	ND	ND	ND	ND	ND	
	4	640	640	640	640	640	
	3	160	640	320	320	320	
	2	80	640	320	80	160	
	1	20	80	20	160	20	
**rgHuN-NP_K357Q_**	6	ND	ND	ND	ND	ND	2.3
	5	320	640	160	320	640	
	4	320	640	320	640	160	
	3	320	160	160	80	10	
	2	80	10	10	20	80	
	1	<10	<10	<10	10	20	
**PBS**		<10	<10	<10	<10	<10	

Notes: Serum were collected from each survival mouse (#1 to #5, respectively) at 14 dpi, and were tested for heamagglutination inhibition (HI) titres against the A/Hunan/42443/2015 virus. Median infectious dose (MID50) was determined using the Karber Method [52]. ND: Not determined due to death of the animal (treated as positive for determination of the MID50). HI titre < 40 was regarded as negative for seroconversion.

As shown in Figure 5(B), the rgHuN_K357Q_ group mice only died when inoculated with 10^6^ TCID_50_ virus. Therefore, the MLD_50_ of the rgHuN_K357Q_ virus was 10^5.5^ TCID_50_. In the rgHuN-WT group, 5/5, 5/5, and 0/5 mice died when inoculated with 10^6^, 10^5^, and 10^4^ TCID_50_ virus, respectively, resulting in an MLD_50_ value of 10^4.5^ TCID_50_. Therefore, the substitution NP-K357Q reduced the virulence of the EA SIVs in mice.

To assess the contribution of the NP-Q357K substitution to the replication of EA SIVs in the respiratory tracts of mice, we inoculated mice with 10^5^ TCID_50_ of each virus and analysed the viral titres in the nasal turbinate, tracheal tissue, and lung at various times after inoculation (Figure 5(C)). The rgHuN viruses with or without the 357K residue in the NP protein both replicated efficiently in the respiratory tracts of the mice. However, the titre of the rgHuN_K357Q_ virus was significantly lower than that of the rgHuN-WT virus in the tracheal tissues of mice at 1, 4, and 7 dpi, and lung tissues of mice at 1 and 4 dpi (*p *< .05, Figure 5(C)). These results indicate that NP-Q357K increases the viral replication of EA SIVs in mice.

### NP-357K viruses exhibited higher polymerase activity and viral growth titres than NP-357Q mutants in human cell lines

To understand whether Q357K substitution affect the viral polymerase activity or virus replications *in vitro*, we firstly performed polymerase activities assay. The result showed that the polymerase complex containing the NP-357K residue showed RNP activity 1.7-fold higher than that of the polymerase complex containing the NP-357Q residue (Figure 5(D)).

We then created two pairs of reverse genetic viruses containing either 357K or 357Q in NP protein in the HuN or JS1 backbone. Based on the growth kinetics in Supplementary Figure 3, back substitution to 357Q in the NP of HuN virus significantly decreased virus replication in human A549 cells (*p *< .05) but not in porcine PK15 cultures. Conversely, introduction of 357K into NP of the JS1 virus enhanced virus replication in human A549 cell lines (48-60 hpi), but this positive effect was not observed in porcine PK15 cells.

## Discussion

EA SIV H1N1 viruses were reported to be the dominantly circulated viruses in pigs in China and have acquired the ability to infect human [6]. Two genotypes of EA H1N1 SIVs have been detected in humans in China, with the HuN-like viruses displaying significantly higher virulence than the JS1-like viruses (Figure 1). Many viral genetic factors are reportedly associated with enhanced virulence, especially the polymerase genes [22,23]. In this study, we found that the NP gene is the major determinant of the virulence of EA H1N1 SIVs. Furthermore, when compared to that of rgHuN-WT, the occurrence of a single substitution V313F or K305R increased viral pathogenicity in mice, while K357Q obviously reduced the viral pathogenicity. Dual mutations K305R + V313F increased the fatality of the viruses. However, the introduction of the K305R into dual mutation K305R + V313F led to a similar degree of viral pathogenicity as rgHuN-WT (Figure 4). Thus, the NP-Q357K mutation in the EA SIVs is fully responsible for the enhanced pathogenicity phenotype, and enhanced the polymerase activity, replication, and infectivity of the viruses in mice.

NP primarily maintains the RNP conformation [41,42], interacting with PB1, PB2, and another NP protein, and plays a central role in the life cycles of the influenza viruses. NP molecules contain a head domain, a body domain, and a protruding tail loop [43,44]. A previous study found that the substitution of N319K in NP protein was located on the surface at the right-hand side of the nucleoprotein body domain and resulted in an increase of polymerase activity [45]. NP-357 was located on RNA-binding groove [46], which contained many basic amino acids and could mediate nucleoprotein–polymerase interactions that are crucial for viral RNA replication [47,48] (Supplementary Figure 4). The introduction of neutral amino acid Q to basic amino acid K substitution might increase the binding of NP proteins to RNAs, and may therefore induce the increased polymerase activity and replication properties (Figure 5(D)) of EA H1N1 viruses. The substitution of NP-Q357K could also enhance pathogenicity of avian H5N1 virus in mice [49].

Q357K in the NP protein is also a typical mammalian-adaptive molecular marker in the influenza A viruses (Figure 3 and Supplementary Table 3). Viruses circulating in birds and humans carry NP-357Q and NP-357K, respectively, whereas among the influenza A viruses circulating in pigs, the majority of CS H1N1 viruses contain NP-357K and most EA H1N1 viruses contain NP-357Q. EA SIVs with NP-357K replicated comparably to those with NP-357Q viruses in porcine cell, but exhibited significantly better growth advantages than NP-357Q viruses in human cell lines (Supplementary Figure 3). In addition, the human-like amino acid mutants NP-Q357K was reported to be likely required for transmission of the 1918 precursor virus to humans [50,51]. Therefore, the NP-Q357K substitution was already present when the avian influenza viruses circulated in pigs, allowing the viruses to infect humans. Viruses with NP-357K more readily circulate in humans than those with NP-357Q.

**Figure 6.**
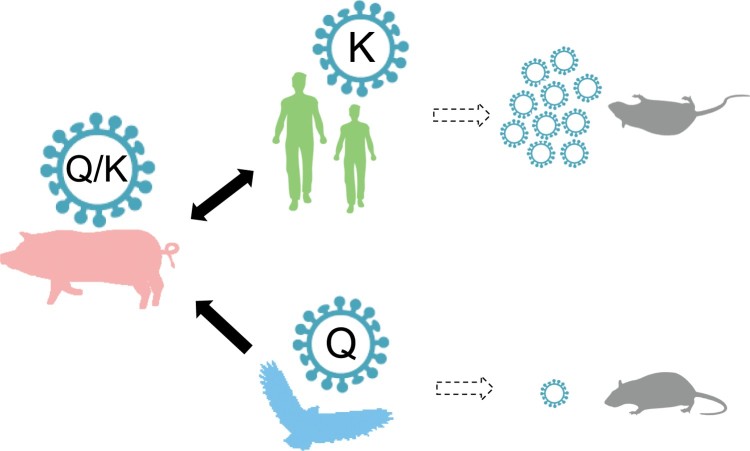
Conceptual model of the generalized inferred evolutionary pathways and virulence outcomes of NP-Q357K in influenza A viruses. Avian influenza viruses carrying NP-357Q were transmitted to pigs. The Q357K substitution was introduced into the NP protein during the adaptation of the avian influenza virus to swine. Mutants with NP-357K were selected, then transmitted to and circulated in humans. The Q357K substitution alters the viral phenotype in mice.

Taken together, our findings suggest that the mutation Q357K in NP is an adaptive signature of the influenza A viruses, allowing them to cross species barriers and altering the viral virulence phenotype in mice (Figure 6). NP-357Q is prevalent in avian viruses, and mutated to NP-357K in the swine population. Viruses with the NP-357K residue were successfully selected and crossed the species barrier to circulate in humans. The mutation of 357Q to 357K increases viral replication and induces a fatal infection in mice. Therefore, because EA H1N1 SIVs have acquired the trait necessary to cause a human influenza pandemic, EA SIVs with NP-Q357K pose a greater risk than those without NP-Q357K, and is a matter of great concern.

## Supplementary Material

Supplemental Material

## References

[1] Castrucci MR, Donatelli I, Sidoli L, et al. Genetic reassortment between avian and human influenza A viruses in Italian pigs. Virology. 1993;193:503–506. doi: 10.1006/viro.1993.1155 8438586

[2] Ma W, Kahn RE, Richt JA. The pig as a mixing vessel for influenza viruses: human and veterinary implications. J Mol Genet Med. 2008;3:158–166. 19565018 PMC2702078

[3] Smith GJ, Bahl J, Vijaykrishna D, et al. Dating the emergence of pandemic influenza viruses. Proc Natl Acad Sci U S A. 2009;106:11709–11712. doi: 10.1073/pnas.0904991106 19597152 PMC2709671

[4] Brown IH. The epidemiology and evolution of influenza viruses in pigs. Vet Microbiol. 2000;74:29–46. doi: 10.1016/S0378-1135(00)00164-4 10799776

[5] Vijaykrishna D, Smith GJ, Pybus OG, et al. Long-term evolution and transmission dynamics of swine influenza A virus. Nature. 2011;473:519–522. doi: 10.1038/nature10004 21614079

[6] Yang H, Chen Y, Qiao C, et al. Prevalence, genetics, and transmissibility in ferrets of Eurasian avian-like H1N1 swine influenza viruses. Proc Natl Acad Sci U S A. 2015. doi:10.1073/pnas.1522643113 PMC4720320 26711995

[7] Liang H, Lam TT, Fan X, et al. Expansion of genotypic diversity and establishment of 2009 H1N1 pandemic-origin internal genes in pigs in China. J Virol. 2014;88:10864–10874. doi: 10.1128/JVI.01327-14 25008935 PMC4178866

[8] Vijaykrishna D, Poon LL, Zhu HC, et al. Reassortment of pandemic H1N1/2009 influenza A virus in swine. Science. 2010;328:1529. doi: 10.1126/science.1189132 20558710 PMC3569847

[9] Zhu WF, Shu YL. [An overview of swine influenza virus infection in humans]. Bing Du Xue Bao. 2013;29:559–565. 24386847

[10] Olsen CW, Brown IH, Easterday BC, et al. Swine influenza. In: Straw B, D’Allaire S, Zimmerman J, Taylor D, editor. Diseases of swine. Iowa, USA: Iowa State University Press; 2005 p. 469–482.

[11] Qi X, Cui L, Jiao Y, et al. Antigenic and genetic characterization of a European avian-like H1N1 swine influenza virus from a boy in China in 2011. Arch Virol. 2012;158:39–53. doi: 10.1007/s00705-012-1423-7 22935945

[12] Wang DY, Qi SX, Li XY, et al. Human infection with Eurasian avian-like influenza A(H1N1) virus, China. Emerg Infect Dis. 2013;19:1709–1711. doi: 10.3201/eid1910.130420 24050834 PMC3810748

[13] Zhu W, Zhang H, Xiang X, et al. Reassortant Eurasian avian-like influenza A(H1N1) virus from a severely Ill Child, Hunan Province, China, 2015. Emerg Infect Dis. 2016;22:1930–1936. doi: 10.3201/eid2211.160181 27767007 PMC5088044

[14] Zhang Y, Zhang Q, Kong H, et al. H5n1 hybrid viruses bearing 2009/H1N1 virus genes transmit in Guinea pigs by respiratory droplet. Science. 2013;340:1459–1463. doi: 10.1126/science.1229455 23641061

[15] Zhou B, Li Y, Halpin R, et al. PB2 residue 158 is a pathogenic determinant of pandemic H1N1 and H5 influenza a viruses in mice. J Virol. 2011;85:357–365. doi: 10.1128/JVI.01694-10 20962098 PMC3014153

[16] Bussey KA, Bousse T, Desmet EA, et al. PB2 residue 271 plays a key role in enhanced polymerase activity of influenza A viruses in mammalian host cells. J Virol. 2010;84:4395–4406. doi: 10.1128/JVI.02642-09 20181719 PMC2863787

[17] Chen H, Bright RA, Subbarao K, et al. Polygenic virulence factors involved in pathogenesis of 1997 Hong Kong H5N1 influenza viruses in mice. Virus Res. 2007;128:159–163. doi: 10.1016/j.virusres.2007.04.017 17521765

[18] Lee MS, Deng MC, Lin YJ, et al. Characterization of an H5N1 avian influenza virus from Taiwan. Vet Microbiol. 2007;124:193–201. doi: 10.1016/j.vetmic.2007.04.021 17512143

[19] Katz JM, Lu X, Tumpey TM, et al. Molecular correlates of influenza A H5N1 virus pathogenesis in mice. J Virol. 2000;74:10807–10810. doi: 10.1128/JVI.74.22.10807-10810.2000 11044127 PMC110957

[20] Conenello GM, Zamarin D, Perrone LA, et al. A single mutation in the PB1-F2 of H5N1 (HK/97) and 1918 influenza A viruses contributes to increased virulence. PLos Pathog. 2007;3:1414–1421. doi: 10.1371/journal.ppat.0030141 17922571 PMC2000966

[21] Subbarao K, Shaw MW. Molecular aspects of avian influenza (H5N1) viruses isolated from humans. Rev Med Virol. 2000;10:337–348. doi: 10.1002/1099-1654(200009/10)10:5<337::AID-RMV292>3.0.CO;2-V 11015744

[22] Hatta M, Gao P, Halfmann P, et al. Molecular basis for high virulence of Hong Kong H5N1 influenza A viruses. Science. 2001;293:1840–1842. doi: 10.1126/science.1062882 11546875

[23] Chen H, Bright RA, Subbarao K, et al. Polygenic virulence factors involved in pathogenesis of 1997 Hong Kong H5N1 influenza viruses in mice. Virus Res. 2007;128:159–163. doi: 10.1016/j.virusres.2007.04.017 17521765

[24] Labadie K DSAE, Rameix-Welti MA, van der Werf S, et al. Host-range determinants on the PB2 protein of influenza A viruses control the interaction between the viral polymerase and nucleoprotein in human cells. Virology. 2007;362:271–282. doi: 10.1016/j.virol.2006.12.027 17270230

[25] Naffakh N, Massin P, Escriou N, et al. Genetic analysis of the compatibility between polymerase proteins from human and avian strains of influenza A viruses. J Gen Virol. 2000;81:1283–1291. doi: 10.1099/0022-1317-81-5-1283 10769071

[26] Munster VJ, de Wit E, van Riel D, et al. The molecular basis of the pathogenicity of the Dutch highly pathogenic human influenza A H7N7 viruses. J Infect Dis. 2007;196:258–265. doi: 10.1086/518792 17570113

[27] Subbarao EK, London W, Murphy BR. A single amino acid in the PB2 gene of influenza A virus is a determinant of host range. J Virol. 1993;67:1761–1764. 8445709 10.1128/jvi.67.4.1761-1764.1993 PMC240216

[28] Hatta M, Gao P, Halfmann P, et al. Molecular Basis for high virulence of Hong Kong H5N1 influenza A viruses. Science. 2001;293:1840–1842. doi: 10.1126/science.1062882 11546875

[29] Zhu W, Li L, Yan Z, et al. Dual E627 K and D701N mutations in the PB2 protein of A(H7N9) influenza virus increased its virulence in mammalian models. Sci Rep. 2015;5:14170. doi: 10.1038/srep14170 26391278 PMC4585756

[30] Gabriel G, Herwig A, Klenk HD. Interaction of polymerase subunit PB2 and NP with importin alpha1 is a determinant of host range of influenza A virus. PLos Pathog. 2008;4:e11. doi: 10.1371/journal.ppat.0040011 18248089 PMC2222953

[31] Le QM, Sakai-Tagawa Y, Ozawa M, et al. Selection of H5N1 influenza virus PB2 during replication in humans. J Virol. 2009;83:5278–5281. doi: 10.1128/JVI.00063-09 19264775 PMC2682078

[32] Li J, Ishaq M, Prudence M, et al. Single mutation at the amino acid position 627 of PB2 that leads to increased virulence of an H5N1 avian influenza virus during adaptation in mice can be compensated by multiple mutations at other sites of PB2. Virus Res. 2009;144:123–129. doi: 10.1016/j.virusres.2009.04.008 19393699

[33] Steel J, Lowen AC, Mubareka S, et al. Transmission of influenza virus in a mammalian host is increased by PB2 amino acids 627 K or 627E/701N. PLos Pathog. 2009;5:e1000252. doi: 10.1371/journal.ppat.1000252 19119420 PMC2603332

[34] Fornek JL, Gillim-Ross L, Santos C, et al. A single-amino-acid substitution in a polymerase protein of an H5N1 influenza virus is associated with systemic infection and impaired T-cell activation in mice. J Virol. 2009;83:11102–11115. doi: 10.1128/JVI.00994-09 19692471 PMC2772766

[35] Gabriel G, Dauber B, Wolff T, et al. The viral polymerase mediates adaptation of an avian influenza virus to a mammalian host. Proc Natl Acad Sci U S A. 2005;102:18590–18595. doi: 10.1073/pnas.0507415102 16339318 PMC1317936

[36] Gao Y, Zhang Y, Shinya K, et al. Identification of amino acids in HA and PB2 critical for the transmission of H5N1 avian influenza viruses in a mammalian host. PLos Pathog. 2009;5:e1000709. doi: 10.1371/journal.ppat.1000709 20041223 PMC2791199

[37] Steel J, Lowen AC, Mubareka S, et al. Transmission of influenza virus in a mammalian host is increased by PB2 amino acids 627 K or 627E/701N. PLoS Pathog. 2009;5:e1000252. doi: 10.1371/journal.ppat.1000252 19119420 PMC2603332

[38] Reed LJ, Skiadopoulos MH. A Simple method of estimating fifty percent endpoints. Am J Hyg. 1938;27:493–497.

[39] Katoh K, Standley DM. MAFFT multiple sequence alignment software version 7: improvements in performance and usability. Mol Biol Evol. 2013;30:772–780. doi: 10.1093/molbev/mst010 23329690 PMC3603318

[40] Kumar S, Stecher G, Tamura K. MEGA7: molecular evolutionary genetics analysis version 7.0 for bigger datasets. Mol Biol Evol. 2016;33:1870–1874. doi: 10.1093/molbev/msw054 27004904 PMC8210823

[41] Yamanaka K, Ishihama A, Nagata K. Reconstitution of influenza virus RNA-nucleoprotein complexes structurally resembling native viral ribonucleoprotein cores. J Biol Chem. 1990;265:11151–11155. 2358455

[42] Ruigrok RW, Baudin F. Structure of influenza virus ribonucleoprotein particles. II. Purified RNA-free influenza virus ribonucleoprotein forms structures that are indistinguishable from the intact influenza virus ribonucleoprotein particles. J Gen Virol. 1995;76(Pt 4):1009–1014. doi: 10.1099/0022-1317-76-4-1009 9049350

[43] Chenavas S, Crepin T, Delmas B, et al. Influenza virus nucleoprotein: structure, RNA binding, oligomerization and antiviral drug target. Future Microbiol. 2013;8:1537–1545. doi: 10.2217/fmb.13.128 24266354

[44] Ye Q, Guu TS, Mata DA, et al. Biochemical and structural evidence in support of a coherent model for the formation of the double-helical influenza A virus ribonucleoprotein. MBio. 2012;4:e00467–e00412. doi: 10.1128/mBio.00467-12 23269829 PMC3531806

[45] Gabriel G, Dauber B, Wolff T, et al. The viral polymerase mediates adaptation of an avian influenza virus to a mammalian host. Proc Natl Acad Sci U S A. 2005;102:18590–18595. doi: 10.1073/pnas.0507415102 16339318 PMC1317936

[46] Ye Q, Krug RM, Tao YJ. The mechanism by which influenza A virus nucleoprotein forms oligomers and binds RNA. Nature. 2006;444:1078–1082. doi: 10.1038/nature05379 17151603

[47] Moeller A, Kirchdoerfer RN, Potter CS, et al. Organization of the influenza virus replication machinery. Science. 2012;338:1631–1634. doi: 10.1126/science.1227270 23180774 PMC3578580

[48] Biswas SK, Boutz PL, Nayak DP. Influenza virus nucleoprotein interacts with influenza virus polymerase proteins. J Virol. 1998;72:5493–5501. 9621005 10.1128/jvi.72.7.5493-5501.1998 PMC110190

[49] Kim JH, Hatta M, Watanabe S, et al. Role of host-specific amino acids in the pathogenicity of avian H5N1 influenza viruses in mice. J Gen Virol. 2010;91:1284–1289. doi: 10.1099/vir.0.018143-0 20016035 PMC2878586

[50] Finkelstein DB, Mukatira S, Mehta PK, et al. Persistent host markers in pandemic and H5N1 influenza viruses. J Virol. 2007;81:10292–10299. doi: 10.1128/JVI.00921-07 17652405 PMC2045501

[51] Manz B, Dornfeld D, Gotz V, et al. Pandemic influenza A viruses escape from restriction by human MxA through adaptive mutations in the nucleoprotein. PLoS Pathog. 2013;9:e1003279. doi: 10.1371/journal.ppat.1003279 23555271 PMC3610643

[52] Karber G. 50% end point calculation. Archiv für Experimentelle Pathologie und Pharmakologie. 1931;162:480–483. doi: 10.1007/BF01863914

