# Supplementary material for: Mammalian-adaptive mutation NP-Q357K in Eurasian H1N1 Swine Influenza viruses determines the virulence phenotype in mice
Source: Emerg Microbes Infect. 2019 Jul 3;8(1):989–99. doi: 10.1080/22221751.2019.1635873 (PMC6609330; doi:10.1080/22221751.2019.1635873)
Supplement: Supplemental Material [file TEMI_A_1635873_SM7113.zip › Supplementary_tables.docx]

**Supplementary table 1. Information on the reverse genetic viruses in this study.**

| **No.** | **Virus name** | **Virus rescue** | **Virus titer** |
| --- | --- | --- | --- |
| 1 | rgHuN-WT | **+** | *** |
| 2 | rgHuN-PB2_JS1_ | **+** | *** |
| 3 | rgHuN-PB1_JS1_ | **+** | *** |
| 4 | rgHuN-PA_JS1_ | **+** | *** |
| 5 | rgHuN-NP_JS1_ | **+** | *** |
| 6 | rgHuN-HA_JS1_ | **+** | *** |
| 7 | rgHuN-NA_JS1_ | **+** | *** |
| 8 | rgHuN-M_JS1_ | **+** | *** |
| 9 | rgHuN-NS_JS1_ | **+** | *** |
| 10 | rgHuN-NP_K305R_ | **+** | *** |
| 11 | rgHuN-NP_V313F_ | **+** | *** |
| 12 | rgHuN-NP_K357Q_ | **+** | *** |
| 13 | rgHuN-NP_K305R+V313F_ | **+** | *** |
| 14 | rgHuN-NP_K305R+K357Q_ | **+** | *** |
| 15 | rgHuN-NP_V313F+K357Q_ | **+** | *** |
| 16 | rgHuN-NP_V313F+K305R+K357Q_ | **+** | *** |
| 17 | rgJS1-WT | **+** | * |
| 18 | rgJS1-PB2_HuN_ | **-** | **-** |
| 19 | rgJS1-PB1_HuN_ | **-** | **-** |
| 20 | rgJS1-PA_HuN_ | **-** | **-** |
| 21 | rgJS1-NP_HuN_ | **+** | ** |
| 22 | rgJS1-HA_HuN_ | **+** | ** |
| 23 | rgJS1-NA_HuN_ | **-** | - |
| 24 | rgJS1-M_HuN_ | **+** | ** |
| 25 | rgJS1-NS_HuN_ | **+** | ** |
| 26 | rgJS1-NP_K305R_ | **-** | - |
| 27 | rgJS1-NP_V313F_ | **-** | - |
| 28 | rgJS1-NP_K357Q_ | **+** | ** |
| 29 | rgJS1-NP_K305R+V313F_ | **-** | - |
| 30 | rgJS1-NP_K305R+K357Q_ | **+** | * |
| 31 | rgJS1-NP_V313F+K357Q_ | **+** | * |
| 32 | rgJS1-NP_V313F+K305R+K357Q_ | **-** | - |

+, viruses were successfully rescued. -, virus was not rescued.

***, virus titer was higher than 10^6^TCID_50_/50ul; **, virus titer was during 10^5^TCID_50_/50ul ~ 10^6^TCID_50_/50ul; *, virus titer was lower than 10^5^TCID_50_/50ul.

**Supplementary table 2 Amino acids differences in NP proteins among JS1-like and HuN-like viruses.**

| Virus | 21 | 48 | 95 | 98 | 99 | 100 | 114 | 119 | 136 | 186 | 189 | 190 | 197 | 217 | 284 | 289 | 305 | 309 | 313 | 323 | 329 | 350 | 353 | 357 | 371 | 377 | 384 | 400 | 401 | 423 | 425 | 430 | 433 | 444 | 452 | 456 | 498 |
| --- | --- | --- | --- | --- | --- | --- | --- | --- | --- | --- | --- | --- | --- | --- | --- | --- | --- | --- | --- | --- | --- | --- | --- | --- | --- | --- | --- | --- | --- | --- | --- | --- | --- | --- | --- | --- | --- |
| A/Jiangsu/1/2011 | N | Q | P | K | K | R | D | I | L | V | M | V | I | I | V | Y | R | S | F | V | I | T | V | Q | M | I | K | R | S | T | I | T | T | I | R | V | N |
| A/Hebei-Yuhua/SWL1250/2012 | . | . | . | . | . | . | . | . | . | . | . | . | . | . | . | . | . | . | . | . | . | . | . | . | . | . | . | . | . | . | . | . | . | . | . | . | . |
| A/Hunan/42443/2015 | D | K | S | R | R | I | E | V | I | I | I | A | . | V | A | H | K | N | V | A | V | K | I | K | V | N | R | K | A | A | V | S | N | V | K | L | S |
| A/Yunnan-Longyang/SWL1982/2015 | D | K | S | R | R | I | E | V | I | I | I | A | V | V | A | H | K | N | V | A | V | K | I | K | V | N | R | K | A | S | V | S | N | V | K | L | S |
| A/Yunnan-Wuhua/SWL1869/2015 | D | K | S | R | R | I | E | V | I | I | I | A | . | V | A | H | K | N | V | A | V | K | I | K | V | N | R | K | T | A | V | S | N | V | K | L | S |

Dots stands for the consistence amino acids to NP sequences of JS1 viruses.

**Supplementary table 3. Database search for amino acids in NP-305, 313 or 357 of influenza A viruses**

| **Amino acid position** | **Hosts** | **Total no. sequences** | **No. same as HuN** | **No. same as JS1** | **No. different** |
| --- | --- | --- | --- | --- | --- |
| 305 | Human | 13834 | 13523 (K, 97.8%) | 309 (R, 2.2%) | 2(L) |
|  | Avian | 12418 | 130 (K, 1.0%) | 12279 (R, 98.9%) | 9(H, C, S) |
|  | Swine | 4322 | 3432 (K, 79.4%) | 725 (R, 16.8%) | 165(N) |
| 313 | Human | 13834 | 4565 (V, 33.0%) | 304 (F, 2.2%) | 8929 (Y), 36 (I, L, H) |
|  | Avian | 12418 | 11 (V, 0.1%) | 12300 (F, 99%) | 107 (L, S, Y, I) |
|  | Swine | 4322 | 1371 (V, 31.7%) | 2861 (F, 66.2%) | 90 (Y, I, L, S, C) |
| 357 | Human | 13834 | 13499 (K, 97.6%) | 258 (Q, 1.9%) | 77 (R) |
|  | Avian | 12418 | 52 (K, 0.4%) | 12357 (Q, 99.5%) | 9 (R, L, H, T) |
|  | Swine | 4322 | 3634 (K, 84.1%) | 624 (Q, 14.4%) | 64 (R, E) |

Polymorphisms of NP-305, 313 and 357 in influenza A viruses were assessed and compared with our findings. The data shown are the number of sequences of different influenza hosts in the NCBI and GISAID database, the number of sequences of identical same amino acid composition as HuN or JS1 (with the amino acids and proportion shown in parentheses), and the number of sequences with a different amino acid at the same position. HuN, A/Hunan/42443/2015 (H1N1); JS1, A/Jiangsu/1/2011 (H1N1).
